# Current status of the use of validated home blood pressure monitoring devices among Korean patients with hypertension

**DOI:** 10.1038/s41440-025-02364-z

**Published:** 2025-09-03

**Authors:** Jieun Lee, Yu-Mi Kim, Seungwon Yoo, Dae-Young Kim, Sung-Hee Shin, Jin Oh Na, Jinho Shin, Kiho Park, Eun Mi Lee

**Affiliations:** 1 Division of Cardiology, Department of Internal Medicine, Korea University Guro Hospital, Seoul, Republic of Korea; 2 Department of Preventive Medicine, Hanyang University College of Medicine, Seoul, Republic of Korea; 3 Mirabellsoft Co., Ltd., Seoul, Republic of Korea; 4 Division of Cardiology, Department of Internal Medicine, Inha University Hospital, Incheon, Republic of Korea; 5 Division of Cardiology, Department of Internal Medicine, Hanyang University College of Medicine, Seoul, Republic of Korea; 6 Division of Cardiology, Department of Internal Medicine, Wonkwang University Sanbon Hospital, Gunpo, Republic of Korea

**Keywords:** Blood Pressure Monitors, Home Blood Pressure Monitoring, Hypertension, Implemental Hypertension, Validated devices

## Abstract

The implementation of Home Blood Pressure Monitoring (HBPM) has improved the diagnosis and treatment of hypertension. Current clinical guidelines advocate the use of validated HBPM devices. However, few studies have examined the current use of validated HBPM devices. We aimed to investigate the current status of the use of validated HBPM devices in real-world hypertensive patients. This study was conducted on Korean patients with hypertension using *CareforMe*®, a smartphone healthcare application which allows the patients to record their HBPM data which can be shared with their physicians. The validation status of HBPM devices was identified based on four registries: STRIDE BP, Medaval, the Japanese Society of Hypertension, and dabl® Educational Trust. From January 2022 through August 2024, 2731 patients entered the model numbers of their HBPM devices. 56.4% (*n* = 1539) of the population were male, and the mean age was 53.5 ± 10.8 years old. 110 models from 33 different manufacturers were identified. 97.0% (*n* = 2649) of the patients used upper-arm devices and 3.0% (*n* = 82) used wrist devices. Based on the four registries, 32.7% (*n* = 36) out of 110 devices were validated, and 51.5% (*n* = 1407) out of 2731 patients used validated devices. Patients using validated devices tend to use more expensive devices than those using non-validated devices (*P* < 0.0001). Among Korean patients with hypertension undergoing HBPM, 97.0% of the patients used upper-arm devices and 51.5% of the patients used validated devices. Our results proposed the urgent need for patient education and public accessibility to validated devices for the better management of patients with hypertension.

**Figure:**
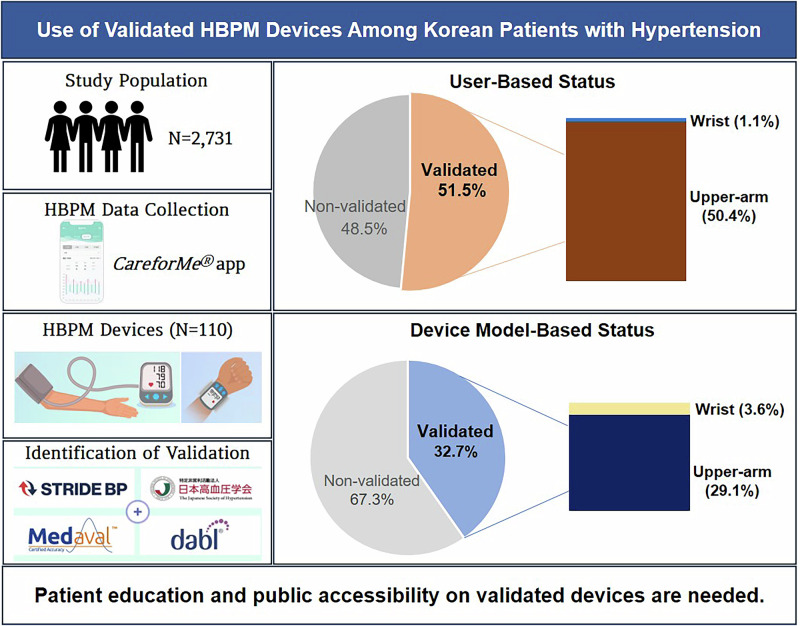
The validation status of HBPM devices was identified based on four registries: STRIDE BP, Medaval, the JSH, and dabl® Educational Trust. 51.5% (*n* = 1,407) out of 2,731 patients used validated devices and 32.7% (*n* = 36) out of 110 devices were validated. Patient education and public accessibility on validated devices are urgently needed. HBPM home blood pressure monitoring; JSH Japanese Society of Hypertension

The validation status of HBPM devices was identified based on four registries: STRIDE BP, Medaval, the JSH, and dabl® Educational Trust. 51.5% (*n* = 1,407) out of 2,731 patients used validated devices and 32.7% (*n* = 36) out of 110 devices were validated. Patient education and public accessibility on validated devices are urgently needed. HBPM home blood pressure monitoring; JSH Japanese Society of Hypertension

## Introduction

Hypertension is the most important modifiable risk factor for cardiovascular diseases causing atherosclerosis and major organ damage [1]. In Korea, 28.0% of adults aged 20 and older are affected by hypertension, resulting in approximately 12.3 million people with hypertension [2]. However, the control rate (Systolic Blood Pressure [SBP] < 140 mmHg or Diastolic Blood Pressure [DBP] < 90 mmHg) among patients with hypertension in Korea is only 58.6% [3]. Efforts to increase the control rate have faced several obstacles, including inaccurate blood pressure (BP) measurements, lack of awareness, therapeutic inertia, and nonadherence to treatment [4–6].

Office BP measurement is essential in the diagnosis and treatment of hypertension [7]; however, it has several limitations including failure to capture BP variations [8], inability to differentiate BP phenotypes, and the potential of mistreatment in individuals with white-coat or masked hypertension. These limitations have led to the complementary use of out-of-office BP measurements such as 24-h ambulatory BP monitoring (ABPM) and home BP monitoring (HBPM). HBPM has several advantages, including its ease of use, repeated measurement capability, cost-effectiveness, and long-term BP monitoring ability in individuals with hypertension. Consequently, HBPM aids in identifying BP phenotypes, predicting cardiovascular morbidity and mortality more effectively than office BP measurements, and improving BP control rates [8–11]. Therefore, reliable devices and standardized techniques are essential to avoid the overestimation or underestimation of home BP.

Current clinical guidelines recommend the use of validated HBPM devices [7, 12–14], and lists of validated BP devices have been provided by various hypertension societies and organizations [15]. However, in most countries, a new BP measurement device does not require validation or a guarantee of its clinical accuracy before being released into the market. Furthermore, only a small number of automated upper-arm and wrist devices globally have evidence of validation [16–18]. In addition, few studies have examined the current use of validated HBPM devices in Korea, and these have been conducted in hospital settings with a small number of patients and in limited regions [19, 20]. Therefore, to ensure the proper use of accurate HBPM devices, it is necessary to assess their validation status in larger populations in various regions and different healthcare settings.

We aimed to investigate the current status of validated HBPM device use among a large population of real-world patients with hypertension treated in hospitals and clinics across different regions in Korea.

Point of View

- Clinical relevance
  It is crucial to improve the current validation status of HBPM devices to help patients measure their home BP accurately to better BP control for minimizing their cardiovascular risks.
- Future direction
  Accredited websites that allow not only physicians but also patients to easily assess and check the validation status of HBPM devices are needed to enhance the public accessibility of validated devices for accurate HBPM and better management of hypertension.
- Consideration for the Asian population
  There is a pressing need to improve the validation status of HBPM devices through the concerted efforts of Asian countries.

## Methods

### Study population

Adult patients aged ≥20 years and diagnosed with hypertension were eligible for enrollment. Individuals who did not use smartphones were excluded from the study. A total of 3002 patients were enrolled between January 2022 and August 2024. (Supplemental Fig. 1). A total of 2731 patients were eligible for analysis, after excluding 206 patients with missing data on HBPM devices or SBP values and 65 patients using kiosk devices. This study was approved by the Institutional Review Board of Wonkwang University Sanbon Hospital (No: WMCSB 202404-04), and all patients signed a written informed consent. This study was conducted in accordance with the ethical principles of the Declaration of Helsinki.

### Home BP data collection

*CareforMe*® is a smartphone healthcare application in which patients can input their HBPM data enabling physicians to see them. Patients were asked to input their age, sex, and information of the medical institutions where they were receiving treatment (a hospital or a clinic, and the region where the medical institution was located) along with their HBPM data including the model numbers and manufacturers of the HBPM devices. The region was classified as capital (Seoul) or non-capital, when the medical institution was not located in Seoul.

### Validation status of the HBPM devices

HBPM devices included upper-arm and wrist devices. The validation status of the HBPM devices was identified according to different general and regional registries: STRIDE BP, Medical device assessment and validation (Medaval, https://medaval.ie/blood-pressure-monitors/), the dabl® Educational Trust, and the Japanese Society of Hypertension [21, 22]. For STRIDE BP, we defined devices which are Validated (devices that have passed established validation procedures), Preferred (devices with at least one STRIDE BP-approved validation study), and Equivalent (equivalent to a device that has fulfilled the criteria for validated devices) or Identical devices as validated devices. HBPM devices were classified as validated in Medaval when the device was recommended with the Medaval assessment for clinical validation. The recommended devices which met the validation requirements of the dabl® Educational Trust were defined as validated devices in the dabl® Educational Trust. HBPM devices found to be validated or equivalent in the JSH were considered validated devices according to the JSH. In this study, we defined validated devices as HBPM devices found to be validated in at least one of these four registries. The authors of this study have checked validation status of each HBPM device based on these four registries. The price of each HBPM device was determined based on the online market.

### Statistical analysis

Continuous variables were summarized using means and standard deviations, while categorical variables were presented as frequencies and proportions. The use of a validated device for each characteristic was assessed using independent t-tests for continuous variables and chi-square tests for categorical variables. A significance level (α) of 0.05 was applied. All data management and analyses were performed using SAS software, version 9.4.

## Results

### Baseline characteristics

Baseline characteristics of the study population (*n* = 2731) are shown in Table 1. 56.4% (*n* = 1539) of the population were male, and the mean age was 53.5 ± 10.8 years old. Patients were enrolled from all over South Korea including the capital (15.5%, *n* = 368). Three university hospitals and 162 primary clinics were identified. A total of 24.8% (*n *= 676) of the patients were treated at hospitals, while 72.8% (*n* = 1989) of the population were receiving treatment at primary clinics. In total, 110 HBPM models from 33 different manufacturers were identified. 97.0% (*n* = 2649) of the patients used upper-arm devices and 3.0% (*n* = 82) were using wrist devices. 55.0% (*n* = 1478) of the patients were using HBPM devices priced 35–70 USD.

**Table 1:** Patient characteristics overall and according to HBPM device validation status

Variables	Total (*n* = 2731)	Validated devices (*n* = 1407)	Non-validated devices (*n* = 1324)	*P* value
Age				
Mean (mean ± SD) (years)	53.5 ± 10.8	53.4 ± 10.9	53.5 ± 10.6	0.892
20–39 years, *n* (%)	251 (9.2%)	128 (9.1%)	123 (9.3%)	0.985
40–64 years, *n* (%)	2,065 (75.6%)	1065 (75.7%)	1000 (75.5%)	
≥65 years, *n* (%)	415 (15.2%)	214 (15.2%)	201 (15.2%)	
Men, *n* (%)	1539 (56.4%)	789 (56.1%)	750 (56.7%)	0.764
Region, n (%)				
Capital (Seoul)	368 (15.5%)	203 (14.4%)	165 (12.5%)	0.133
Non-capital	2,363 (86.5%)	1204 (85.6%)	1159 (87.5%)	
Type of medical institution, *n* (%)				
Hospital	676 (24.8%)	333 (24.2%)	343 (26.6%)	0.167
Clinic	1,989 (72.8%)	1041 (75.8%)	948 (73.4%)	
Missing data	66 (2.4%)	33 (2.3%)	33 (2.5%)	
Mean duration of *CareforMe*^®^ use (days)	328.8 ± 258.9	326.6 ± 260.0	331.2 ± 257.8	0.636
Patients checking HBPM ≥ 1 time/week, *n* (%)	1803 (66.0%)	927 (65.9%)	881 (66.5%)	0.717
Type of HBPM devices, *n* (%)				
Upper-arm devices	2649 (97.0%)	1377 (97.9%)	1272 (96.1%)	0.006
Wrist devices	82 (3.0%)	30 (2.1%)	52 (3.9%)	
HBPM devices based on price, *n* (%)				
0–35 USD	341 (12.7%)	121 (8.6%)	220 (16.6%)	<0.0001
35–70 USD	1478 (55.0%)	582 (41.4%)	896 (67.7%)	
Expensive than 70 USD	867 (32.3%)	699 (49.7%)	168 (12.7%)	
Price information not available	45 (1.6%)	5 (0.4%)	40 (3.0%)	

*CareforMe*® is a smartphone healthcare application in which patients can input their HBPM data enabling physicians to see them. Data are shown for the total population and for subgroups using validated vs. non-validated devices. *P*-values represent comparisons between validated and non-validated device users

*HBPM* home blood pressure monitoring, *USD* United States dollar

### Validation status of the HBPM devices

**Fig. 1:**
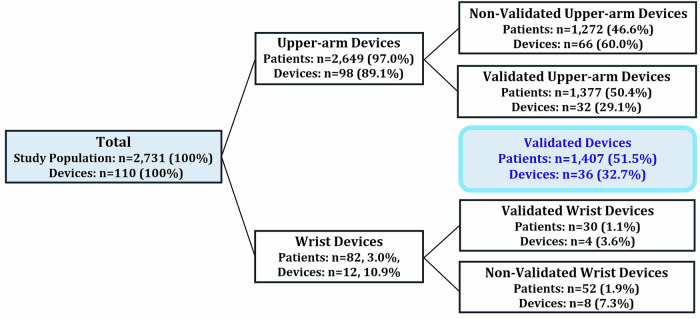
Use of Validated HBPM Devices According to Patients and Devices. Out of 2731 patients (110 devices), 1407 (51.5%) used validated devices (devices: *n* = 36, 32.7%), including 1377 (50.4%) using validated upper-arm devices (devices: *n* = 32, 29.1%) and 30 patients (1.1%) using validated wrist devices (devices: 4, 3.6%). HBPM home blood pressure monitoring

Table 2 shows the validation status of the HBPM devices according to different registries. Among the four registries, validation status was highest (number of people: 46.0%, *n* = 1255, number of devices: 22.7%, *n* = 25) with STRIDE BP, while showing the lowest validation status with JSH registry (number of people: 6.4%, *n* = 174, number of devices: 7.3%, *n* = 8). When the four registries were combined, 32.7% (*n* = 36) out of 110 devices were validated, and 51.5% (*n* = 1407) out of 2731 patients were using validated devices (Fig. 1).

**Table 2:** Validation status of HBPM devices based on four registries

	STRIDE BP	Medaval	JSH	dabl	Total (STRIDE BP + Medaval + JSH + dabl)
User based (n = 2731), *n* (%)					
Validated	1255 (46.0%)	461 (16.9%)	174 (6.4%)	807 (29.5%)	1407 (51.5%)
Non-validated	1476 (54.0%)	2270(83.1%)	2557 (93.6%)	1924 (70.5%)	1324 (48.5%)
Device model based (*n* = 110), *n* (%)					
Validated	25 (22.7%)	16 (14.5%)	8 (7.3%)	25 (22.7%)	36 (32.7%)
Non-validated	85 (77.3%)	94 (85.5%)	102 (92.7%)	85 (77.3%)	74 (67.3%)

*Medaval* Medical device assessment and validation, *JSH* Japanese Society of Hypertension, *dabl* dabl® Educational Trust

Patient characteristics according to the HBPM devices are presented in Table 1. There were no significant differences between patients using validated devices and those using non-validated devices according to age and sex. The proportion of the patients treated in the capital was higher in patients using validated devices although it was not statistically significant (14.4% vs. 12.5%; *P* = 0.133). The proportion of patients using upper-arm devices was higher in those using validated devices than in those using non-validated devices (97.9% vs. 96.1%, *P* = 0.006). Patients using validated devices used more expensive devices than those using non-validated devices (*P* < 0.0001).

We categorized the HBPM devices according to their manufacturers and analyzed the 3 main manufacturers chosen by the patients (Table 3). Most patients using validated devices (1217 out of 1407 patients) used HBPM devices from these three manufacturers.

**Table 3:** HBPM devices of the top 3 most used manufacturers

Manufacturers	Patents, *n* (%)	Validation Status	Patents, *n* (%)	No. of Models, *n* (%)	Validation Status	No. of Models, *n* (%)
A	1529 (56.0%)	Validated	811 (53.0%)	29 (26.4%)	Validated	16 (55.2%)
		Non-Validated	718 (47.0%)		Non-Validated	13(44.8%)
B	338 (12.4%)	Validated	335 (99.1%)	3 (2.7%)	Validated	2 (66.7%)
		Non-Validated	3 (0.9%)		Non-Validated	1 (33.3%)
C	210 (7.7%)	Validated	71 (33.8%)	8 (7.3%)	Validated	2 (25.0%)
		Non-Validated	139 (66.2%)		Non-Validated	6 (75.0%)

## Discussion

Among Korean patients with hypertension undergoing HBPM, 97.0% of the patients used upper-arm devices, while 3.0% used wrist devices. Based on the four registries, 32.7% of the devices were validated. Overall, 51.5% of the patients were using validated devices, including 50.4% of upper-arm device users and 1.1% of wrist device users. To the best of our knowledge, this is the first large-scale, multi-center study to evaluate the current status of the use of validated HBPM devices among Korean patients with hypertension.

HBPM devices are automated instruments which enable users to measure their BP with a simple pressing of a button [14]. However, automated devices have limitations. These devices detect arterial oscillations and estimate the mean BP when the oscillation amplitude is maximal, subsequently calculate BP indirectly using proprietary algorithms known only by the manufacturer. Because these algorithms are not interchangeable among different devices and are not publicly disclosed, they should be tested rigorously on clinical accuracy and precision of devices against a reference standard with globally established protocols, known as validation [15]. The use of non-validated devices could undermine optimal medical practice through misdiagnosis and inappropriate management of hypertension [11, 23].

Currently, formal clinical validation of BP devices is not mandatory in all countries to be put on the market. In 2020, approximately 38% of the HBPM devices in the world were validated [17]. Few studies have been conducted regarding the current use of validated HBPM devices. An Australian study found out that only 18.3% of the upper-arm devices and 8.0% of wrist devices were found to be validated [18]. A study based on the Medaval database showed that approximately 19.9% of the devices were validated or equivalent [16]. There were two Korean regional studies about validated BP devices. Jung et al. conducted a single-center study of 212 patients and found that 38.7% of the patients used validated upper-arm devices [19]. Recently, Rhee et al. [20] conducted a survey study in two cities (number of patients = 359) and found that 52.0% of the patients were using validated upper-arm devices. As such, the validation status of HBPM devices varies between studies, and the current status of the use of validated HBPM devices is unknown. The validation status of HBPM devices in our study differs from that of two previous Korean regional studies [19, 20], although all three studies showed a higher validation status of HBPM devices in Korea than in other countries.

The validation status of HBPM devices can differ between studies according to the validation protocols adapted by the researchers. Jung et al. [19] conducted a study in a tertiary cardiology outpatient clinic located in Suwon on 212 patients assessed the validation status of HBPM devices brought to the clinic by the patients based on dabl® Educational Trust and British Hypertension Society websites. The community-based study by Rhee et al. [20], which included 673 participants residing in Goyang and Paju assessed the validation status of HBPM devices provided as pictures by participants, based on the websites (STRIDE BP, US Blood Pressure Validate Device Listing, JSH, and Medaval) and queries to the manufacturers. Our study could represent real-world data of Korean patients with hypertension because of the large number of patients comprising more heterogeneous and general populations from various regions and healthcare settings. Furthermore, we have checked the validation status of each HBPM device based on the four renowned global and regional registries, with accurate model numbers of the HBPM devices provided by the patients.

Over the past 30 years, various scientific societies and associations have developed validation protocols for BP devices, but fewer than 10% of devices have been published established protocols [7]. Several registries are operated by national or continental scientific societies, including the Japanese Society of Hypertension (JSH), STRIDE BP (affiliated with the International Society of Hypertension, European Society of Hypertension, and the World Hypertension League), Medaval (affiliated with the World Hypertension League), and the Dabl® Educational Trust (the first widely used device registry established by a group of international investigators) [24]. Internationally established validation protocols include the Association for the Advancement of Medical Instrumentation (AAMI), the American National Standards Institute (ANSI), the British Hypertension Society (BHS), the European Society of Hypertension International Protocol (ESH-IP), and the International Organization for Standardization Universal Standard (ISO). Each registry has its own lists of validated devices according to accepted validation protocols [15]: STRIDE BP accepts AAMI/ ANSI/ ISO, BHS, and ESH-IP; Medaval includes AAMI/ ANSI/ ISO, BHS, ESH-IP, and German Hypertension League Quality Seal Protocol; Dabl® Educational Trust uses AAMI, BHS, and ESH-IP; and JSH lists devices submitted by manufacturers. Because of the variety of internationally established validation protocols and accepted validation protocols by each registry, we assessed the validation status of HBPM devices across multiple global and regional registries.

Even though South Korea has the highest treatment and control rates in the world [3, 25], there are currently no Korean websites or associations on which patients can find validated devices. As our study is the first large-scale, multi-center study to analyze the current status of the use of validated HBPM devices among real-world hypertensive patients in Korea including a large number of patients with heterogeneous backgrounds and precise data, we sincerely hope that our study could become an initiative for improving the validation status of HBPM devices to help patients measure their home BP more accurately to control their BP better for minimizing their cardiovascular risks. It would be ideal but impossible to conduct accredited validation testing for all BP monitors. Nevertheless, encouraging the endorsement of clinically validated devices can help manufacturers to care more about clinical validation for accuracy as well as device safety. Furthermore, we strongly agree with an international statement of developing a single universally accredited list of BP monitors [13, 26–28] in the future for accurate BP measurement and better control of hypertension. Accredited websites that allow not only physicians but also patients to easily assess and check the validation status of HBPM devices are needed to enhance the public accessibility of validated devices for accurate HBPM and better management of hypertension.

This study has several limitations. First, the population using *CareforMe*® were included in this study. Although this study included a large number of patients across South Korea, selection bias may have been present because we did not apply formal sampling procedure among the population according to the region. Second, the socioeconomic status of each patient could affect the type of HBPM device used because our study found that patients using validated devices tended to use more expensive devices than patients using non-validated devices.

### Perspective of Asia

While few studies have evaluated the current use of validated HBPM devices, our study holds particular significance as the first large-scale, multi-center investigation to assess their use among real-world Korean patients with hypertension. In Asian populations, blood pressure is more strongly associated with cardiovascular diseases such as stroke and heart failure, and greater benefits from blood pressure reduction have been observed compared to Western populations [29–32]. However, substantial heterogeneity in hypertension awareness and control rates exists across Asian countries, largely due to differences in healthcare infrastructure, public health strategies, and socioeconomic conditions. In this context, the use of clinically validated HBPM devices is essential for improving hypertension diagnosis and management in Asia. Despite these needs, our study reveals a low rate of validated device use in actual clinical practice, highlighting the urgent need to improve the validation status and accessibility of HBPM devices through coordinated efforts across Asian countries.

## Conclusions

To the best of our knowledge, this is the first study to analyze the current status of the use of validated HBPM devices among hypertensive patients in Korea. Among Korean patients with hypertension undergoing HBPM, 97.0% of the patients used upper-arm devices and 51.5% of the patients were using validated devices. Our results proposed the urgent need for patient education on accurate HBPM and public accessibility to validated devices for better management of patients with hypertension.

## Supplementary information

Supplemental Figure 1

Supplemental Figure 1 Legend
