# Supplementary figures and images for: Current status of the use of validated home blood pressure monitoring devices among Korean patients with hypertension
Source: Hypertens Res. 2025 Sep 3;48(11):2811–8. doi: 10.1038/s41440-025-02364-z (PMC12586138; doi:10.1038/s41440-025-02364-z)

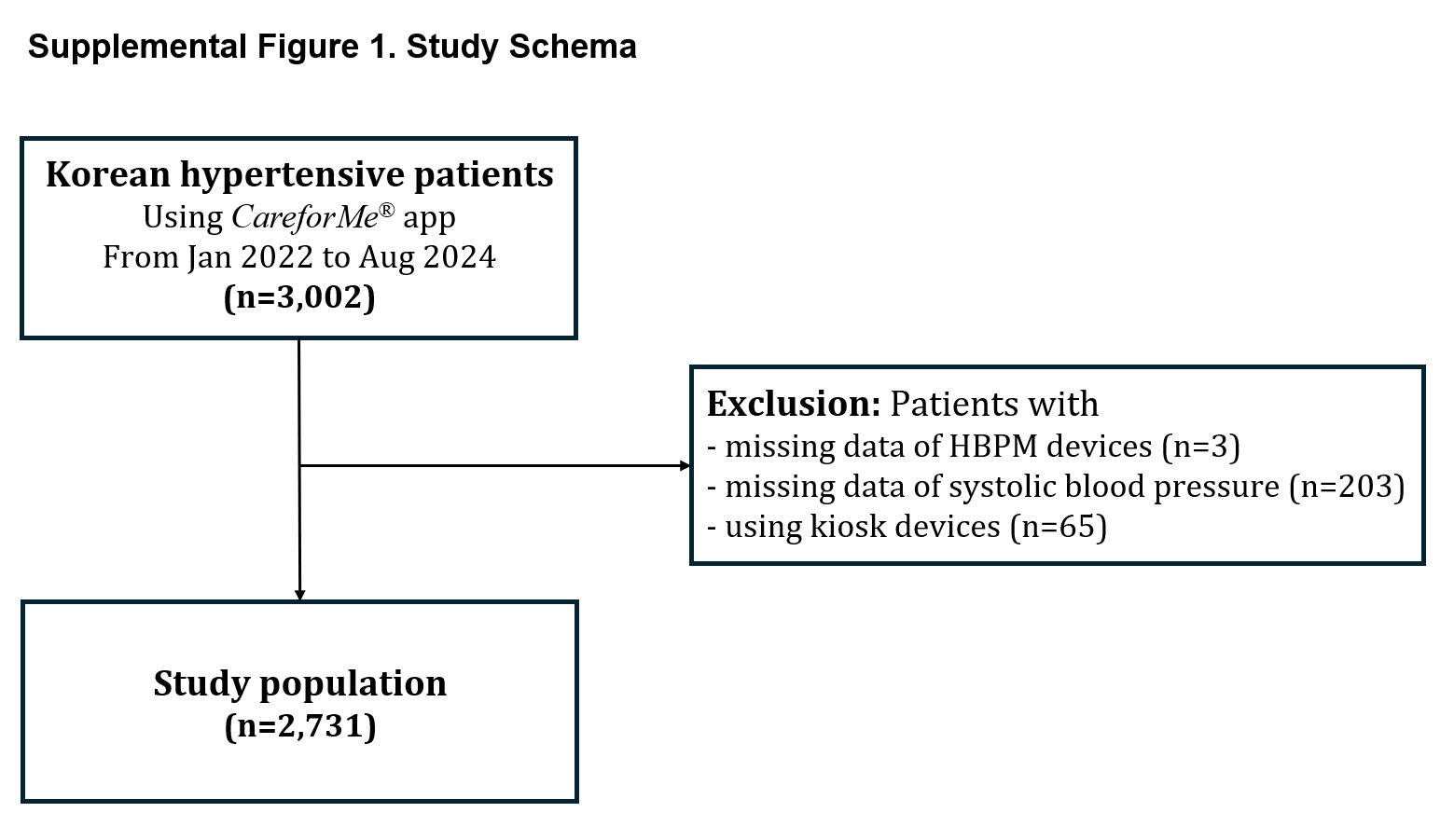

Supplement: Supplementary file 1 — Supplemental Figure 1 [file 41440_2025_2364_MOESM1_ESM.jpg]
