# Supplementary material for: Current status of the use of validated home blood pressure monitoring devices among Korean patients with hypertension
Source: Hypertens Res. 2025 Sep 3;48(11):2811–8. doi: 10.1038/s41440-025-02364-z (PMC12586138; doi:10.1038/s41440-025-02364-z)
Supplement: Supplementary file 2 — Supplemental Figure 1 Legend [file 41440_2025_2364_MOESM2_ESM.docx]

**Supplemental Figure 1. Study Schema**

Among the 3,002 patients enrolled between January 2022 and August 2024, a total of 2,731 patients were eligible for analysis after excluding 206 patients with missing data on HBPM devices or SBP and 65 patients using kiosk devices.

HBPM = home blood pressure monitoring; SBP = systolic blood pressure
